# Supplementary material for: The interactome of intact mitochondria by cross-linking mass spectrometry provides evidence for coexisting respiratory supercomplexes
Source: Mol Cell Proteomics. 2017 Dec 8;17(2):216–32. doi: 10.1074/mcp.RA117.000470 (PMC5795388; doi:10.1074/mcp.RA117.000470)
Supplement: Supplemental Data [file supp_RA117.000470_133922_0_supp_23978_fzffwf.zip › spectra_annotation/mito_DR_spectra_annotation/494-1-3-1-10-1.pdf]

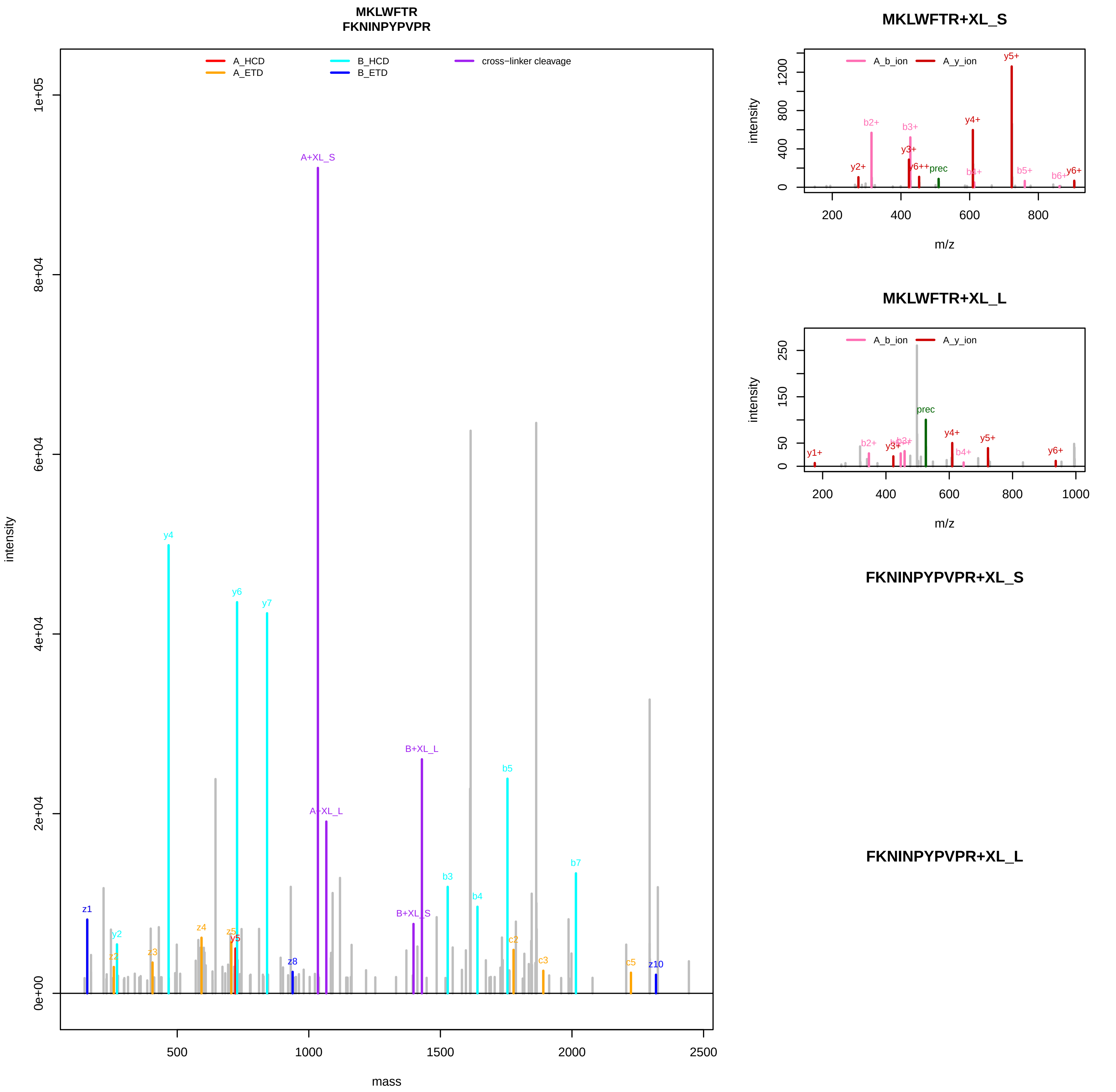

MKLWFTR
FKNINPYPVPR
A_HCD
A_ETD
B_HCD
B_ETD
cross−linker cleavage
A+XL_S
A+XL_L
B+XL_L
B+XL_S
y2
y4
y5
y6
y7
z1
z2
z3
z4
z5
z8
z10
b3
b4
b5
b7
c2
c3
c5
intensity
mass
0e+00
2e+04
4e+04
6e+04
8e+04
1e+05
500
1000
1500
2000
2500
MKLWFTR+XL_S
A_b_ion
A_y_ion
b2+
b3+
b4+
b5+
b6+
y2+
y3+
y4+
y5+
y6+
y6++
prec
intensity
m/z
0
400
800
1200
200
400
600
800
MKLWFTR+XL_L
A_b_ion
A_y_ion
y1+
b2+
y3+
b4+
y4+
y5+
y6+
prec
intensity
m/z
0
50
150
250
200
400
600
800
1000
FKNINPYPVPR+XL_S
FKNINPYPVPR+XL_L
